# Supplementary material for: Understanding glioblastoma invasion using physically-guided neural networks with internal variables
Source: PLoS Comput Biol. 2022 Apr 4;18(4):e1010019. doi: 10.1371/journal.pcbi.1010019 (PMC9009781; doi:10.1371/journal.pcbi.1010019)
Supplement: Attachment — Submitted filename: Review.pdf [file pcbi.1010019.s001.pdf]

Review of manuscript PCOMPBIOL-D-21-01502

SUMMARY

The paper is generally well written and deals with an interesting problem, both from the point of view of heterogeneity of glioblastoma, as well as the methodology which has the potential to learn biologically relevant information from data.

The paper is concerned with the application of the PGNNIV framework (Physically-guided neural networks with internal variables) to study the go-or-grow hypothesis for glioblastoma which postulates that cells switch between migratory and proliferative phenotypes. In the current work it is assumed to be driven by oxygen concentration. The data used is synthetically generated using a PDE model previously used to model the evolution of cancer cell density and oxygen concentration in a microfluidic device.

The strength of PGNNIV framework, which has been published previously, is that it can both be used to make predictions as well as learn relationships related to non-measurable variables. In the current case these are two functions which governs how oxygen concentration influences advective velocity and proliferation rates of the cell population.

The authors claim that the presented method is more flexible than previous frameworks (PINNs and BINNs), as well as generally performing better than standard parametric learning frameworks both in terms of predicting future cell culture evolution as well as learning the unknown functions governing the go-or-grow mechanism. The former could benefit from a more thorough discussion/motivation, and the latter claim is indeed demonstrated for a number of different in-silico experimental conditions. From the discussion and general theme of the paper, focus is on understanding glioblastoma, however due to the lack of experimental data specific conclusions cannot be drawn.

Following is a list of comments and questions, ranging from major to minor, followed by a list of typos.

MAJOR

1. The work is interesting and the method appears promising, however the results would be greatly improved and method's utility more believable if it was applied to experimental data. The title suggests that the understanding of glioblastoma invasion is furthered, and mechanisms are unraveled, although no data is present, and no conclusions regarding glioblastoma were drawn.

2. The go or grow hypothesis is often postulated as migration and proliferation being spatiotemporally exclusive processes. There are many instances where this has been modelled using two subpopulations, one representing cells which only migrate and one representing cells which only proliferate (e.g. Fedotov and Iomin PRL 2007, Gerlee and Nelander PLOS Computational Biology 2012, Stepien et al. Siam J. Appl. Math. 2018). In such models the switching terms between either state has an easily interpretable meaning, namely the rate at which cells change phenotype. Is there any deeper interpretation of the two functions Π_{go} and Π_{gr}, in particular in the case that their sum is not 1?

As a follow-up question, what is the motivation for assuming that the go-term represented by Π_{go} only influences the advection term and not the random motility term? Since therapeutic evaluations are discussed, the random motility would presumably be of equal importance, and in the current framework it is specified beforehand and not inferred from the data. Would not this be a limitation when it comes to generality of the model?

3. It is mentioned that the proposed framework is more flexible compared to PINNs and BINNs. Could you please elaborate on this and explain the differences in greater detail?

4. It would be interesting to know more about the practical aspects, such as the computational demands and the hardware/software used. Is it possible to train your network on the dataset used on a standard PC within reasonable times, or would it require special hardware such as GPUs? It would also be interesting to know if the proposed method is faster or slower than the parametric approach used for comparison.

MINOR

1. On lines 74-82: In my opinion it should be emphasized that the method is used on synthetic data and not experimental data. First time reading the paper I was under the impression at this point that experimental data would be used.

2. Line 275: Here R is used to represent residual, whereas on line 237 and Eq. 8 it is used to describe the physical constraints. This is confusing.

3. Figure 3 is almost unreadable when zooming in. This may be due to formatting of images for review, in that case disregard the comment. If not, it needs to be made larger or increased resolution. Moreover, there is a tiny red underscoring of D_x in Fig 3a that should be removed.

4. The notation of $\hat{u}$ shows up in Fig 3 and Eq 19 but it is not described in the text.

5. Should the arguments of R in Eq. 20 be u^n and $\hat{u}^{n+1}(u_n)$?

6. Line 261-262: What is this function F used in $F(u_{2j})$? Is this at all related to the bold F described previously, or merely used to symbolize an arbitrary relationship?

7. Line 333: A single sentence about the numerical method referenced would be useful to the reader.

8. The description of batch in the section "Feeding the network": Is your use of the word "batch" the traditional one? i.e. the network parameters are updated after each such batch?

9. Regarding the section "Training process". Is the data shuffled at any point (before initiation, between epochs, the order of batches, etc.)?

TYPOS

1. Line 68: "a concept that han been", should read "a concept that has been"?

2. In the caption of Fig 1: "Assumed as unidimensional with length the width of the chamber, l". This must be a typo.

3. Line 183: Remove the three dots inside the parentheses.

4. Line 204: Shouldn't the range of the function be $\mathbb{R}^2$?
5. Line 286: It appears that the sentence was cropped off by mistake: "$v = \Pi$, while $u = (u_1, u_2)$"?
6. Line 345: "whith" should be "with"
7. Line 392: Missing section name between "Section" and "except".
