# Supplementary material for: Understanding glioblastoma invasion using physically-guided neural networks with internal variables
Source: PLoS Comput Biol. 2022 Apr 4;18(4):e1010019. doi: 10.1371/journal.pcbi.1010019 (PMC9009781; doi:10.1371/journal.pcbi.1010019)
Supplement: Attachment — Submitted filename: Letter_To_Reviewers.pdf [file pcbi.1010019.s002.pdf]

First, we would like to thank the reviewer for their comments that have substantially improved the paper. The manuscript has been revised and rewritten in some parts to consider the suggestions of the reviewer.

Next, the response to the reviewers' comments is presented. Reviewer comments are in black bold typeface and the authors' responses in blue normal typeface. When referring to the manuscript, the authors have indicated the added/removed words in **red** color. These changes are marked in the manuscript too.

The paper is generally well written and deals with an interesting problem, both from the point of view of heterogeneity of glioblastoma, as well as the methodology which has the potential to learn biologically relevant information from data.

The paper is concerned with the application of the PGNNIV framework (Physically-guided neural networks with internal variables) to study the go-or-grow hypothesis for glioblastoma which postulates that cells switch between migratory and proliferative phenotypes. In the current work it is assumed to be driven by oxygen concentration. The data used is synthetically generated using a PDE model previously used to model the evolution of cancer cell density and oxygen concentration in a microfluidic device.

The strength of PGNNIV framework, which has been published previously, is that it can both be used to make predictions as well as learn relationships related to non-measurable variables. In the current case these are two functions which governs how oxygen concentration influences advective velocity and proliferation rates of the cell population.

The authors claim that the presented method is more flexible than previous frameworks (PINNs and BINNs), as well as generally performing better than standard parametric learning frameworks both in terms of predicting future cell culture evolution as well as learning the unknown functions governing the go-or-grow mechanism. The former could benefit from a more thorough discussion/motivation, and the latter claim is indeed demonstrated for a number of different in-silico experimental conditions. From the discussion and general theme of the paper, focus is on understanding glioblastoma, however due to the lack of experimental data specific conclusions cannot be drawn.

Following is a list of comments and questions, ranging from major to minor, followed by a list of typos.

MAJOR:

1. The work is interesting and the method appears promising, however the results would be greatly improved and method's utility more believable if it was applied to experimental data. The title suggests that the understanding of glioblastoma invasion is furthered, and mechanisms are unraveled, although no data is present, and no conclusions regarding glioblastoma were drawn.

The reviewer is completely right. Since no experimental data is used throughout the paper, we changed the title to "Understanding glioblastoma invasion using physically-guided neural networks with internal variables". Moreover, both the introduction and conclusions sections have been adapted to insist on the fact that no experimental data have been used in the study.

- In Abstract, we have replaced

  "we apply these techniques"

  by

  "we show how these techniques could be applied"

- At the introduction, we changed **L81 – L84**. Previously it was written:

  "This methodology does not only permit the identification of complex metabolic changes but also improving the prediction accuracy of parametric models."

  And now:

  "The data used for testing the methodology are generated in silico*, thus allowing evaluating the predictive and explanatory capacity of PGNNIV. Indeed, we demonstrate that this methodology does not only permit the identification of complex metabolic changes but also improves the prediction accuracy of parametric models."*

- In the conclusion section, we have changed **L569-L574**. Previously it was written:

  "PGNNIV have demonstrated their unravelling capacity for different benchmark-tested models, thus allowing us to work in model-free and non-parametric frameworks."

  And now:

  "Using in silico *data, we have proven the unravelling capacity of PGNNIV for different benchmark test models, thus allowing us to work in model-free and non-parametric frameworks."*

In addition to the reviewer comment, the main aim and motivation of our paper were to introduce and develop a novel simulation methodology with application to glioblastoma (GBM) invasion. Along the paper, we have highlighted the great potential and applicability of PGNNIV to understand GBM cancer progression and evolution, especially to contrast biological hypotheses, and, eventually, as an alternative to phenomenological modeling. These two unique features make PGNNIV a useful tool in modeling complex biological problems. In this paper, we focused on the ability of PGNNIV to unravel metabolic switch functions, something that was tested using synthetic data obtained from a previously validated mathematical model. The presented approach is a first step usually required before its application to real data, which will require monitoring and acquisition of a large amount of data in microfluidic platforms.

Indeed, we are currently working in our lab on this aspect. Cell culture monitoring in real time and in a continuous fashion is challenging from the technical point of view and requires new and adapted acquisition techniques, surpassing the ones used in previous works published by our group [1,2], that used the cell concentration profiles at some very specific snapshots. Also, the derivation of such an important conclusion, as it is the go-or-grow transition behavior for a given cell line, should be endorsed with further biological evidences and extra experimental tests (for instance, genetic expression tests).

However, we consider this methodology worth of publication in Plos CB given the demonstrated methodological and scientific novelty of the study.

2. The go or grow hypothesis is often postulated as migration and proliferation being spatiotemporally exclusive processes. There are many instances where this has been modelled using two subpopulations, one representing cells which only migrate and one representing cells which only proliferate (e.g. Fedotov and Iomin PRL 2007, Gerlee and Nelander PLOS Computational Biology 2012, Stepien et al. Siam J. Appl. Math. 2018). In such models the switching terms between either state has an easily interpretable meaning, namely the rate at which cells change phenotype.

   The reviewer points out a very interesting question related to the system modeling, and supports their questions using some scientific references. However, when compared to other multi-component models presented in the literature (see the discussion presented in [1], section Mathematical methods/Model equations, where some different models are reviewed), our approach presents several important advantages.

   The model that inspired our approach has only 8 parameters, with many of them well-enough characterized in the literature (α_1, D_2, k_m and θ).

   Previous works such as [1] have demonstrated that with this parametric model we have been able to fit experimental data under different experimental configurations, in comparison with other works, such as those cited by the reviewer, that are of course very interesting from the computational point of view but do not include experimental data.

On the other hand, there are many different examples in the literature considering two or even three distinct phenotypes of GBM populations (see for instance reference [27] in our paper). However, there is no direct biological evidence about such a discrete differentiation between these phenotypes. The use of the smooth *go or grow* approach, guarantees a smooth transition and flexibility between the proliferative and migratory activity. It is true that the model is more easily interpreted using a family of discrete phenotypes, as the reviewer indicates, but our approach seems to be closer to biological evidence where no abrupt transition between proliferative and migratory phenotypes is observed.

Despite these pros, the fundamental reason for selecting this model is for illustrative purposes. The presented methodology's strength relies on the capacity for unravelling hidden internal equations, such as $\mathbf{\Pi} = \mathbf{\Pi}(O_2)$, a continuous function relating the oxygen level with the migratory and proliferative activity. With a multi-component model, the PGNNIV approach is only able to perform parametric fitting (the different transition rates and the motility parameters). Of course, more sophisticated models may include some non-parametric dependencies, but here we have sought a compromise between complexity and interpretability.

Anyway, we have included the following sentence in **L108 - L111**:

"Although some works support the use of two [34-36] or even three [27] phenotypes for describing the cell population, we use a previously validated model offering some flexibility for modeling the switch between proliferative and migratory activity."

Is there any deeper interpretation of the two functions Π_{go} and Π_{gr}, in particular in the case that their sum is not 1?

This discussion is very interesting and is treated briefly in the Discussion section. Under the assumption of two different phenotypes, this function may be difficult to interpret, but in the adopted continuum-phenotype framework, these functions encode the energy resources spent by the cell in a migrate/proliferate process. For instance, and as an illustration, if the proliferation cost is twice the cost of migration (in appropriate dimensionless variables), the balance of energy resources would lead to $2\Pi_{\mathrm{gr}} + \Pi_{\mathrm{go}} = 1$, that is $2\Pi_{\mathrm{gr}} + \Pi_{\mathrm{go}} - 1 = 0$. In general, this energetic constraint must be expressed as $G\left(\Pi_{\mathrm{gr}}, \Pi_{\mathrm{go}}\right) = 0$, for an appropriate function G; something that may be easily enforced within the PGNNIV framework. However, this is essentially a biological discussion while here we focus on the unravelling capacity of the methodology, that does not depend on the relation between Π_{gr} and Π_{go}. Indeed, with the presented framework, the actual relation between Π_{gr} and Π_{go} could be estimated *a posteriori* using the learned models for both functions. The fact that, during the whole paper, the benchmark models used satisfy $\Pi_{\mathrm{gr}} + \Pi_{\mathrm{go}} = 1$ is just a matter of simplicity for reducing the number of parameters. To remark on this aspect, we added the following in **L196 – L198**:

"Indeed, the only biological evidence is that Π_{gr} is a nondecreasing function and Π_{go} is a nonincreasing function. The relation between the two may be, in general, more complex, assumed as unknown, or even be unveiled also by the network."

As a follow-up question, what is the motivation for assuming that the go-term represented by Π_{go} only influences the advection term and not the random motility term? Since therapeutic evaluations are discussed, the random motility would presumably be of equal importance, and in the current framework it is specified beforehand and not inferred from the data. Would not this be a limitation when it comes to generality of the model?

The reviewer points out here an interesting question, which is the fact that our method focuses on the unveiling of the advection term, instead of considering both the advection and the random motility term. This is explained by the following:

- First, our model relies on a previously published model that considers both terms separately. Actually, the cell flux term is:

$$F = D\frac{\partial u}{\partial x} - K_n \Pi_{go}(O_2;\theta) u \frac{\partial O_2}{\partial x},$$

  where:

$$\Pi_{go}(x;\theta) = \begin{cases} 1 \text{ if } x \leq 0 \\ 1 - \frac{x}{\theta} \text{ if } 0 \leq x \leq \theta \\ 1 \text{ if } x > \theta \end{cases}$$

  This is one possible model among a wide range of possibilities (some other possibilities are discussed in the paper), but has shown good agreement with real experimental data. Besides, in Ref. [1], an extensive literature review was performed, justifying the adoption of this kind of decoupling between the random and oxygen-driven migration, rather than adopting, for instance:

$$F = \left(D\frac{\partial u}{\partial x} - K_n u \frac{\partial O_2}{\partial x}\right)\Pi_{go}(O_2;\theta)$$

- However, and regardless of this observation based on biological evidence, this assumption is only for illustration purposes and does not affect at all the presented methodology. The only requirement to adapt the network to this flux structure is changing the network topology according to a new version of Equation (9a):

$$\dot{\boldsymbol{u}}_1 = \boldsymbol{D}\left[(D_1 \boldsymbol{D}\boldsymbol{u}_1 - \chi \boldsymbol{u}_1 \odot \boldsymbol{D}\boldsymbol{u}_2) \odot \boldsymbol{\Pi}_{go}\right] + \alpha_1 \boldsymbol{\Pi}_{gr} \odot \boldsymbol{u}_1 \odot \left(1 - \frac{\boldsymbol{u}_1}{c_s}\right)$$

- Finally, and as a last observation, recall that any model parameter may be declared to be learned during the training process, in addition to the model parameters associated with the model subnetworks (in that case $\boldsymbol{\Pi}_{go}$ and $\boldsymbol{\Pi}_{gr}$). That is, during the training process, we can learn, in addition to the functional state model $\boldsymbol{\Pi} =$

$\mathbf{\Pi}(\boldsymbol{u}_2)$, the values of the parameters $D_1, \chi, \alpha_1, c_s, D_2, \alpha_2$ and k_m. We did not proceed this way as, as pointed out by the reviewer, the data were synthetically generated and the main novelty of this work is the unravelling capacity of non-parametric dependencies rather than fitting specific parameter values, something that of course could be interesting when facing real data. Indeed, and related with the next reviewer comment, the parametric fittings used as a baseline for comparison purposes have been obtained using this strategy.

To clarify these aspects, we added the following at **L271 - L276**:

"In addition to the model $\boldsymbol{\Pi}$, it would be possible to consider the rest of the specific model parameters ($D_1, \chi, \alpha_1, c_s, D_2, \alpha_2$ and k_m) as parameters to be learned during the training process. However, as this is similar to conventional parametric fitting (except for the fact that we use the broad capabilities of NN hardware and software [30]), we consider them here as known, with their values detailed next when specifying data generation."

3. It is mentioned that the proposed framework is more flexible compared to PINNs and BINNs. Could you please elaborate on this and explain the differences in greater detail?

   PGNNIVs improve BINNs (a particular case of PINNs) in at least two aspects:

   - In the BINN framework, the network is formulated as $\boldsymbol{u} = \mathcal{N}(x; \boldsymbol{\Lambda})$, where $\boldsymbol{\Lambda}$ are the network model parameters, some of them related to the submodel that is intended to be unveiled, and the loss function is expressed as $\mathcal{L} = \mathcal{L}_{\text{DATA}} + \mathcal{L}_{\text{PHYSICS}}$. Consequently, once the network is trained, the network is able to predict, for fixed initial and boundary conditions, the value of the field $\boldsymbol{u}(x)$ at a given spatial point x. PGNNIV are more flexible in the sense that their architecture leverages the problem structure to focus their predictive capacity on the internal state model (the function $\mathbf{\Pi}$). This is illustrated in the paper by variating the boundary conditions and simulating the possible experimental outcomes for different initial conditions (normoxic, hypoxic and gradient configuration), after a single training process. The reviewer is right that, perhaps, these ideas are not sufficiently developed in the text, so we have added in **L322 – L328**:

     "Note that the architecture defining the PGNNIV, represented in Fig. 3 and summarized in Eqs. (18) and (20), representing respectively the predictive and explanatory network, does not depend on the initial and boundary conditions. This implies that the network may be trained with data coming from different experimental configurations, thus ensuring that the explanatory network H is properly represented in the data. Once trained, predictions can be

made, for any external stimuli and initial state. This is indeed one of the main differences between BINNs and PGNNIVs."

- Besides, due to its structure, a BINN presents some limitations for considering arbitrary internal state models. The state models are represented by a MLP (or other one input – one output structures) relating two specific neurons. Using automatic differentiation, this could be extended to local operators of order one (even if this case has not been exploited yet and does not appear in [3]). However, for more general operators (higher order local operators, fractional operators, integral operators, nonlinear functionals…), the value of the output depends on the values of the input in a neighborhood of the point or even on the values on the whole domain, so a greater flexibility in the operator definition is necessary. As the PGNNIV work at the nodal value level, this difficulty is overcome by construction. This discussion is interesting but is technical and relies on many mathematical and computational aspects that are maybe out of the scope of a Computational Biology journal. That is why we have decided not to include it in this paper. However, all these details have been published in a paper where we discussed broadly the application of PGNNIV to continuum problems, and that is cited along the text [4]. Anyway, to give more information to the reader, we added at **L71**:

  "such as non-local or global models [31]"

4. It would be interesting to know more about the practical aspects, such as the computational demands and the hardware/software used. Is it possible to train your network on the dataset used on a standard PC within reasonable times, or would it require special hardware such as GPUs? It would also be interesting to know if the proposed method is faster or slower than the parametric approach used for comparison.

   The reviewer is right that this information was missing. According to their comment, we added a new section in the manuscript, "Computational requirements" before the Discussion section. We hope that with this new section, the required practical aspects are now elucidated.

MINOR:

1. On lines 74-82: In my opinion it should be emphasized that the method is used on synthetic data and not experimental data. First time reading the paper I was under the impression at this point that experimental data would be used.

   We have changed these lines, as commented in the first major comment. We hope that now it is clear from the beginning for the reader that this paper focuses on the methodological aspects rather than in the biological particular conclusions, even if this method could be, in principle, applied to any available dataset.

2. Line 275: Here R is used to represent residual, whereas on line 237 and Eq. 8 it is used to describe the physical constraints. This is confusing.

   The notation might seem confusing at first, but this ambiguity disappears once it is observed that, indeed, the physical constraints and the residual are actually identical: the discrete form of the PDEs representing the cell and oxygen evolution encodes all the physical information related to the problem. To clarify this aspect we added the clause *"that is indeed the equation encoding the problem physics"* at **L288**.

3. Figure 3 is almost unreadable when zooming in. This may be due to formatting of images for review, in that case disregard the comment. If not, it needs to be made larger or increased resolution. Moreover, there is a tiny red underscoring of D_x in Fig 3a that should be removed.

   We have improved the quality of all figures. Also, the red underscoring pointed out by the reviewer has been removed.

4. The notation of $\hat{u}$ shows up in Fig 3 and Eq 19 but it is not described in the text.

   The reviewer is right. We forgot to define this notation for the fields predicted value. We added the sentence "*where we have denoted by $\hat{\boldsymbol{u}}_i^{n+1}$ the predicted value of the field $\boldsymbol{u}$ by the PGNNIV*" in **L318**.

5. Should the arguments of R in Eq. 20 be u^n and $\hat{u}^{n+1}(u^n)$?

   The value of $\hat{u}^{n+1}$ depends on u^n, so the dependence of R on $\hat{u}^{n+1}$ is superfluous. We have however explicitly stated the dependency on u^n and u^{n+1} as these are the data that feed the network at each time training step, that is, the field values at two consecutive time-steps for the whole evolution process. To clarify this aspect, we added in **L321**:

   "where we have defined the residual as a function of the data, $\boldsymbol{R} = \boldsymbol{R}(\boldsymbol{u}^n, \boldsymbol{u}^{n+1})$."

6. Line 261-262: What is this function F used in $F(u_{2j})$? Is this at all related to the bold F described previously, or merely used to symbolize an arbitrary relationship?

We thank the reviewer for this observation that we overlooked. We intended to symbolize an arbitrary relationship but the use of the letter F is indeed confusing. We have changed it by the more natural expression $\Pi_j(u_{2j})$.

7. Line 333: A single sentence about the numerical method referenced would be useful to the reader.

   We have considered the reviewer proposal and we added the following sentence in **L357 – L361**:

   "The system of equations was solved numerically by means of a time-space integrator based on a piecewise nonlinear Galerkin approach which is second order accurate in space, and compatible with this kind of nonlinear equations and boundary conditions, using the Matlab `pdepe` *suit."*

8. The description of batch in the section "Feeding the network": Is your use of the word "batch" the traditional one? i.e. the network parameters are updated after each such batch?

   Yes. We have investigated different training strategies, variating the way the neural network is fed using the synthetic experimental data (for instance considering each batch as the whole dataset, by introducing each experimental data series always in the same order or by shuffling them using random sampling, by considering only partial measurements of the series…). The presented one, that is, considering each complete synthetic experiment series as one batch of data and sampling it without replacement, was the one producing the best results, although for other strategies, similar results were obtained. As the paper is focused on the method rather than on the different training strategies, we have reported this final result for clarity and conciseness. We added a few lines clarifying this aspect and the next one (see next comment).

9. Regarding the section "Training process". Is the data shuffled at any point (before initiation, between epochs, the order of batches, etc.)?

   The reviewer is right, this information was missing. To clarify this aspect and, according to the explained in the previous comment, we added the following sentence at **L382-L385**:

   "the $M = 11$ synthetic configurations, each one of them corresponding to one batch of data (so that the network parameters are updated after each batch feed), were sampled without replacement, so they were used in a different order at each epoch iteration."

TYPOS:

They all have been corrected, with respect to the comment:

In the caption of Fig 1: “Assumed as unidimensional with length the width of the chamber,l”. This must be a typo.

Maybe the reviewer referred to the use of unidimensional instead of one-dimensional. This word has been corrected here and all along the paper.

OTHER MINOR CHANGES:

We have read the whole manuscript and corrected the following typos/details:

- The operator $\boldsymbol{D}_{x}$ is now denoted as $\boldsymbol{D}$ for avoiding the confusion between the operator and the generic variable over which it acts in Fig. 3.

- We have slightly modified Fig. 3 for including the correct signs at the operator definition (see the following figure) and also for including the χ parameter. These are minor corrections (as the signs may be included in the parameter’s values, but now the figure is more consistent with the rest of the paper).

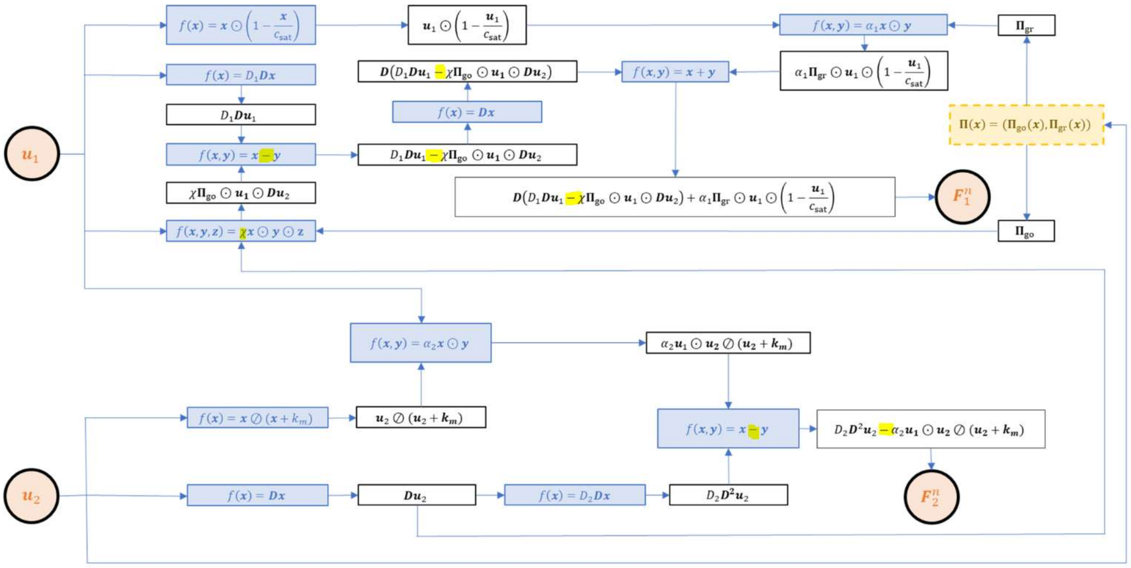

- For consistency purposes, we have proofread the whole manuscript to adapt all the words’ spelling to American English.

REFERENCES

[1] Ayensa-Jiménez, J., Pérez-Aliacar, M., Randelovic, T., Oliván, S., Fernández, L., Sanz-Herrera, J. A., ... & Doblaré, M. (2020). Mathematical formulation and parametric analysis of in vitro cell models in microfluidic devices: application to different stages of glioblastoma evolution. *Scientific Reports*, *10*(1), 1-21.

[2] Pérez-Aliacar, M., Doweidar, M. H., Doblaré, M., & Ayensa-Jiménez, J. (2021). Predicting cell behaviour parameters from glioblastoma on a chip images. A deep learning approach. *Computers in Biology and Medicine*, *135*, 104547.

[3] Lagergren, J. H., Nardini, J. T., Baker, R. E., Simpson, M. J., & Flores, K. B. (2020). Biologically-informed neural networks guide mechanistic modeling from sparse experimental data. *PLoS computational biology*, *16*(12), e1008462.

[4] Ayensa-Jiménez, J., Doweidar, M. H., Sanz-Herrera, J. A., & Doblaré, M. (2020). On the application of Physically-Guided Neural Networks with Internal Variables to Continuum Problems. *arXiv preprint arXiv:2011.11376*.
